# The economic and personal burden of cluster headache: a controlled cross-sectional study

**DOI:** 10.1186/s10194-022-01427-7

**Published:** 2022-05-24

**Authors:** Anja Sofie Petersen, Nunu Lund, Agneta Snoer, Rigmor Højland Jensen, Mads Barloese

**Affiliations:** 1 Danish Headache Center, Department of Neurology, Rigshospitalet-Glostrup, University of Copenhagen, Glostrup, Denmark; 2 Department of Clinical Physiology and Nuclear Medicine, Centre for Functional and Diagnostic Imaging and Research, Hvidovre Hospital, Hvidovre, Denmark

**Keywords:** Burden of disease, Cluster headache, Quality of life, Direct cost, Indirect cost

## Abstract

**Background:**

Cluster headache is a less-prevalent primary headache disorder but is overrepresented with regards to use of health care and social services. More insight into the socioeconomic impact is required.

**Methods:**

We investigated both the personal and societal disease burden and cost in 400 patients with well-classified cluster headache according to the ICHD-criteria and 200 sex- and age matched controls. All participants completed a cross sectional questionnaire and semi-structured interview.

**Results:**

Patients with chronic cluster headache constituted 146 out of 400 (37%). Overall, restriction in personal and/or professional life was reported by 94% of patients during attack periods. Even in remission, nine times as many episodic patients rated their health as poor/very poor compared to controls (9% vs 1%, *p* = 0.002). For chronic patients, the odds of rating health as good/very good were ten times lower compared to controls (OR:10.10, 95%CI:5.29–18.79. *p* < 0.001) and three times lower compared to episodic patients in remission (OR:3.22, 95%CI:1.90–5.47, *p* < 0.001). Additionally, chronic cluster headache patients were 5 times more likely to receive disability pension compared to episodic (OR:5.0, 95%CI:2.3–10.9, *p* < 0.001). The mean direct annual costs amounted to 9,158€ and 2,763€ for chronic and episodic patients, respectively (*p* < 0.001). We identified a substantial loss of productivity due to absence from work resulting in a higher indirect cost of 11,809 €/year/patient in the chronic population and 3,558 €/year/patient in the episodic population. Presenteeism could not be quantified but productivity was reduced in patients by 65% in periods with attacks compared to controls.

**Conclusion:**

Cluster headache has a major negative impact on personal life, self-perceived health, and societal cost. Patients with the chronic variant are vastly more burdened. Patients with the episodic form were still markedly affected during the remission period. This study highlights the need for more effective therapy to lighten the burden on patients and society.

## Background

The majority of the cluster headache (CH) population is episodic (eCH) and experience attacks in bouts, thus being in remission for months to years [1, 2]. The remission period relieves the patients of the severe pain and may also ease known comorbidities, such as depression and anxiety [3]. Yet, it has been reported that avoidance behavior and worries about future attacks continue throughout the remission period [4] and for many patients sleep disturbances persist outside the bout[2, 5]. In addition, CH-related symptoms such as shadow attacks (*a mild pain attack that resembles a CH attack but is short-lasting and less intense*) and solitary attacks are not uncommon during remission [6]. This raises the question whether CH patients in remission can be regarded as “healthy” or as sufferers from a chronic disease with some symptoms manifesting cyclically and others permanently. Therefore, our first hypothesis was that eCH patients in remission were more burdened compared to controls. Anecdotally, CH tends to abate over time [7] but clinically, usually lasts for at least several decades with an early age of onset typically in the third decade [8]. This combination of early onset and long-term disease duration have enormous potential to impact both the individual, with regards to life choices in family and professional matters, but also the society in terms of direct and indirect costs. Per definition, chronic CH (cCH) patients do not go into meaningful remission periods [9] and intuitively this group should be more burdened by the disease. Overall, the literature supports this notion, however, a recent review concludes that the current evidence level is too sparse to draw firm conclusions [10]. Our second hypothesis was therefore that cCH patients were more burdened in personal life and costs compared to eCH patients.

We aimed to explore the impact on both personal and societal parameters to compile a comprehensive assessment of the disease burden in a large cohort of eCH and cCH patients compared to controls.

## Methods

The data was obtained from the Danish Cluster Headache Survey, which is a cross-sectional questionnaire study. Data collection was performed between 2012 and 2017. The publication on the primary analysis describes the methodology in details [2] and succeeding publications [11–16] are based on the same methodology.

### Participants

**Fig. 1:**
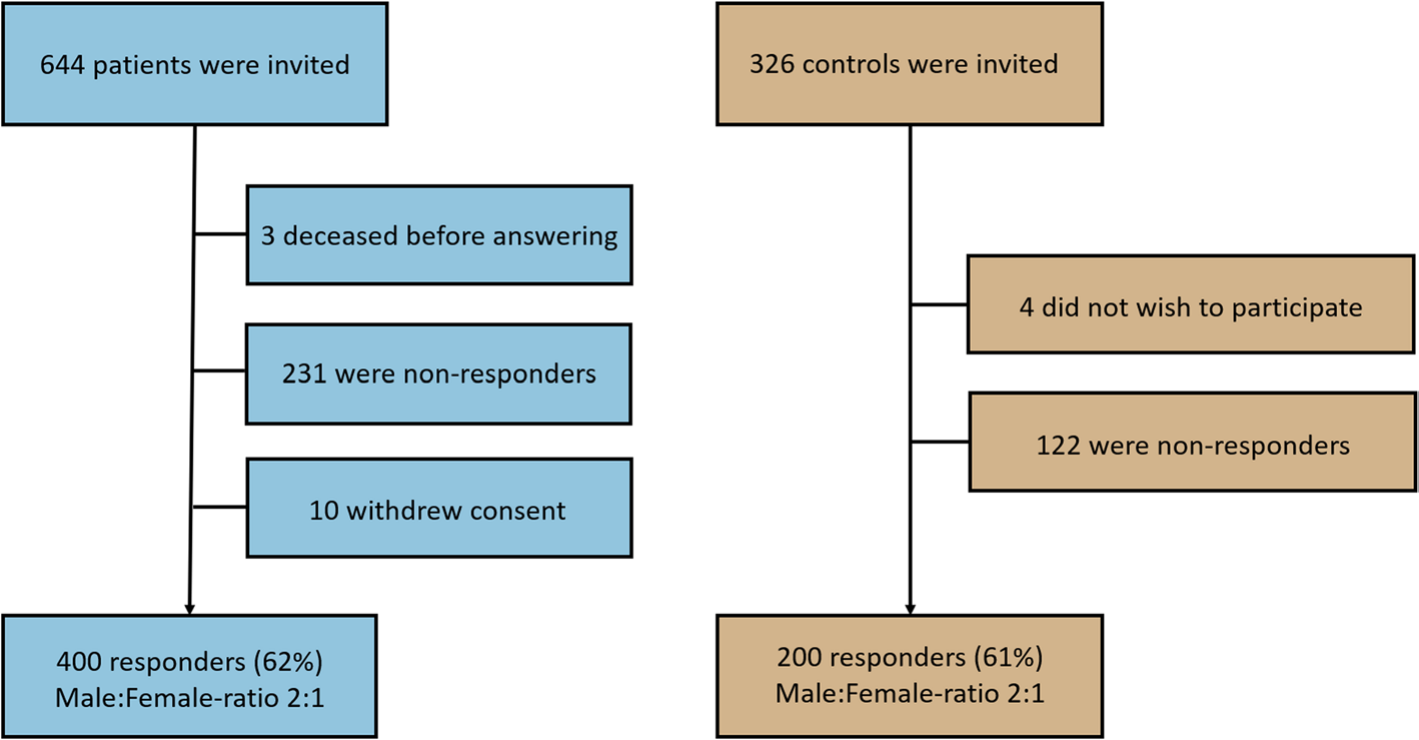
Flowchart of inclusion. Data presented for 400 patients with cluster headache. Means (SD) are based on the entire population, not only the patients receiving the specific healthcare service or social benefit. Underneath the cost the patients who received the service or benefit within the last year are indicated. The costs were not normally distributed, and we therefore applied the non-parametric Wilcoxon test. Some total costs do not total the sum of the means due to rounding

In total 600 participants, 400 CH patients and 200 controls contributed to the study (Fig. 1). Patients were diagnosed with CH according to the International Classification of Headache Disorders (ICHD) II edition and all diagnoses were validated by a headache specialist at the Danish Headache Center. Patients suspected to have an underlying condition causing the CH or having another chronic headache disorder were excluded. Inclusion of a patient with a comorbid episodic headache required ability to distinguish CH attacks from other headache attacks. Recruitment was mainly based at the Danish Headache Center, however, a few patients were recruited from private neurologists and from online advertisements. Controls were matched for age and sex and allowed to have headache up to one day/month. All participants were fluent in Danish and between 18–65 years.

### Questionnaire

The questionnaire consisted of 365 questions that were subdivided into seven sections [2]. Ambiguities and missing data were clarified by the succeeding semi-structured interview by a physician, trained medical student or study nurse (MB, NL, MF, AP).

Regarding the personal burden of CH, we investigated three different domains: social, family and professional life. All questions included in this study were developed by authors MB and RJ based on previous epidemiological headache questionnaires [17, 18]. Self-rated health and wellbeing were rated on a five-level scale: Very good, good, neither poor nor good, poor or very poor. Participants were asked if they received fulltime or part-time disability pension and if they received full-time disability pension, whether it was based on their CH diagnosis. Ability to work was rated on an eleven-point rating scale (0 being not reduced and 10 being completely reduced) in periods with attacks for CH patients and controls were asked to rate their work productivity in general. Patients were asked when they experienced their first attack, and this defined the onset of CH.

### Cost calculation

The direct cost of CH was calculated as the sum of self-reported use of acute medication and healthcare services within the last year. Estimated prices are adjusted for the year 2021 and expressed in Euros. We did not include expenses for prophylactic treatment as this has previously been determined to be a very minor contributor of total costs [19] and during the time of data collection the CGRP-antibodies were not available. For direct cost of acute treatment, only patients with attacks within the last year were included. In Denmark, oxygen is paid for by the hospital, and delivered to the patients through sub-contractors. Currently the cost of oxygen is a fixed daily price. We estimated rental time of the equipment based on bout duration and added 60 days for return in the eCH population. In Denmark, the prices on drugs may vary on a weekly basis. Here, we used the prices for the cheapest option in the 2^nd^ week of March 2021. Number of units used per day was estimated by attack frequency and self-reported percentages of attacks treated with a given treatment.

The indirect cost of illness was calculated following an incidence-based bottom-up approach. In the indirect cost analysis, we included absenteeism (sick days and disability pension) but not presenteeism. Participants could choose not to share their salary. If these patients reported sick days, their salary was assumed equivalent to other patients with the same education level. Participants could indicate if the fulltime disability pension had been approved due to CH, and only patients who indicated this were included in the indirect cost analysis. In Denmark, the compensation rate for part-time disability depends upon the salary and the reduction of working hours. We did not have access to the exact reduction hours and therefore assumed a work week for the part-time disabled of 20 h and an hourly wage of 26.7 € including pension. We could not determine if the part-time disability pension was approved due to CH or to comorbidities, but it is assumed that CH at least in part contributed to the decision.

### Statistics

R 3.6.1 and Rstudio 1.2.5001 were used for statistically analysis. Descriptive data is presented as mean (SD) or as a numeric (percentage) distribution. To compare categorical variables across categories the chi-square test was used and to compare continuous variables we applied one-way ANOVA or a two-sample t-test. Bonferroni correction was applied to the impact on everyday life in Fig. 3. Logistic regression (GLM function in R) was used to investigate the potential association between disability pension and clinical or demographic factors. We explored the literature on sociodemographic and CH specific factors of receiving disability pension and three variables was selected: sex, educational level and phenotype (cCH or eCH). We further explored the relationship between age of CH onset and disability pension because it is potential confounder of highest achieved education. Regarding self-rated health we applied an ordinal logistic regression using the “polr”-function in R. In order to increase power, we dichotomized participants responding poor or very poor and good or very good. We aimed to investigate phenotype on self-rated health, and we chose to control for sex and occupational status. No power calculation was preformed since we already publish the primary sleep analysis.

### Registrations and approval

The Capital Region of Denmark Ethical Committee approved the study (H-2–2012-016) as part of a sleep CH-study [20]. After this study was completed the survey did not need further formal approval from the ethics committee (file-number: 17008910). Patients gave informed oral and written consent according to the Helsinki declaration.

## Results

In total, 600 participants completed both the questionnaire and the semi-structured interview. Response rates were 400 out of 645 (62%) for patients and 200 out of 328 (61%) for controls. 253 participants were diagnosed with eCH and 146 with cCH. At time of interview one patient could not be classified as eCH or cCH (first bout) and was excluded from sub-analyses. Patients in bout, 124 out of 253 eCH patients (49%), were defined as having one or more attacks within the last month. In a previous publication from the same patient group [16] we found that male patients constituted a significantly larger part in the episodic group compared to the chronic group, respectively 182 out of 253 eCH patients (72%) and 86 out of 146 cCH patients (59%). The patients were 46.2 ± 11.5 years and had an average 3.6 ± 2.3 attacks/day. The controls were 44.7 ± 13.1 years and 133 (67%) were male. See Table 1 for further demographics. Pain intensity of usual attacks was rated severe or very severe by 95% of patients. Details regarding sleep, lifestyle, comorbidities, diagnostic factors and treatment have previously been published [12–14, 16].

**Table 1:** Demographics of the study population

	**Episodic cluster headache (*N* = 253)**	**Chronic cluster headache (*N* = 146)**	**Controls** **(*N* = 200)**
**Age, years**	46.5 (11.2)	45.8 (12.2)	44.7 (13.1)
**Sex ratio (Male:Female)**	2.56:1	1.43:1	1.99:1
**Marital status (N, %)**			
Single/unmarried	30 (12%)	14 (10%)	9 (5%)
Married	172 (68%)	102 (70%)	149 (75%)
Divorced/widow(er)	51 (20%)	20 (21%)	21 (21%)
**Highest achieved education (N, %)**			
Student	10 (4%)	7 (5%)	20 (10%)
Primary/secondary	19 (8%)	24 (16%)	8 (4%)
Technical	47 (19%)	21 (14%)	12 (6%)
Trainee	24 (10%)	21 (14%)	11 (6%)
Associate degree	37 (15%)	16 (11%)	15 (8%)
Bachelor degree	53 (21%)	35 (24%)	47 (24%)
Master degree or higher	50 (20%)	14 (10%)	85 (43%)
Other degree	13 (5%)	8 (6%)	2 (1%)
**Current occupation (N, %)**			
Student	10 (4%)	7 (5%)	20 (10%)
Employed (full/part time, self-employed)	162 (63%)	48 (33%)	157 (79%)
Unemployed	18 (7%)	19 (13%)	9 (5%)
Sick leave	13 (5%)	24 (16%)	1 (1%)
Disability pension (full/part time)	34 (13%)	42 (29%)	3 (2%)
Retired	16 (6%)	6 (4%)	10 (5%)
**Household taxable income pr year (N, %)**			
< 66,667 €	113 (45%)	77 (53%)	61 (31%)
66,667–133,333 €	104 (41%)	39 (27%)	76 (38%)
> 133,333 €	14 (6%)	10 (7%)	45 (23%)
Do not wish to share income	22 (9%)	20 (14%)	18 (9%)

Data presented for 400 patients and 200 controls as means (SD) for numerical variables and as number (percentages) for categorical variables

### Impact on self-rated health

**Fig. 2:**
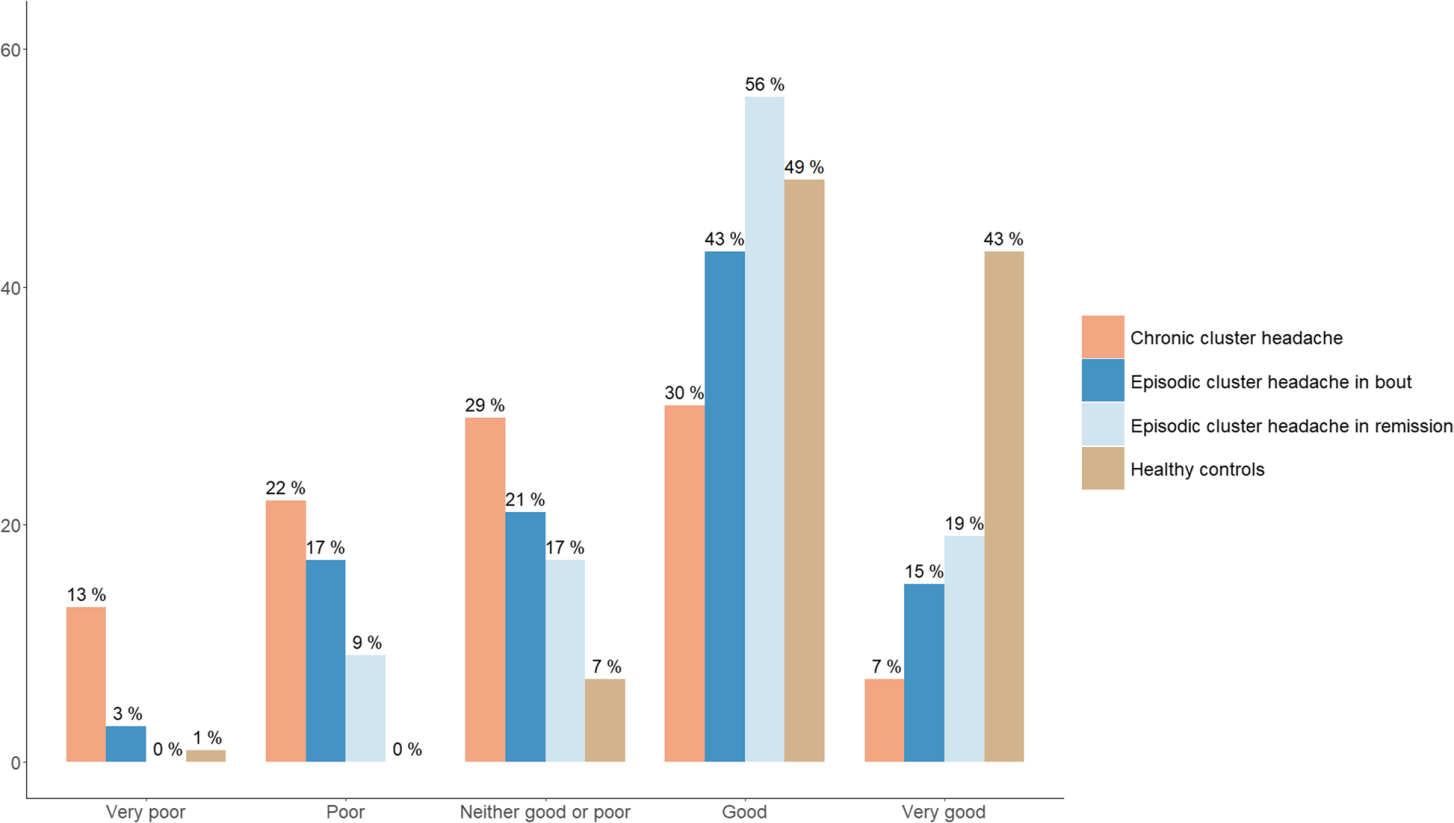
Self-rated health among patients with cluster headache and controls. Legend: Patients and controls were asked to rate their current health (*N* = 599). Patients in bout were defined as having a cluster headache attack within the last month. We dichotomized participants responding poor or very poor as poor and good or very good as good to increase power in an ordinal logistic regression adjusted for occupation and gender. Chronic cluster headache was the reference. Episodic cluster headache in bout were statistically indifferent to the chronic patients (OR 1.57, 95%CI 0.96–2.57, *p* = 0.074), The episodic cluster headache in remission were statistically different from the chronic patients (OR 3.22, 95%CI 1.90–5.47, *p* < 0.001), as were the controls (OR 10.10, 95%CI 5.29–18.79. *p* < 0.001)

Health ratings are visualized in Fig. 2. Health was rated as poor/very poor by one-fifth of the eCH patients in bout compared to more than one-third of the cCH patients (26 out of 127 (20%) vs 49 out of 144 (34%), *p* = 0.0009). Only, 2 (1%) of the controls and 11 (9%) of the eCH patients in remission rated their health as poor/very poor (*p* = 0.002). In general, self-rated wellbeing followed the same pattern as self-rated health. In those 57 eCH patients who had been in remission over one year only 3 (5%) reported their health as poor/very poor.

Compared to a cCH patient, the odds to rate health as good/very good were 3 times higher for an eCH patient in remission (OR 3.22, 95%CI 1.90–5.47, *p* < 0.001) and 10 times higher for a control (OR 10.10, 95%CI 5.29–18.79. *p* < 0.001) when correcting for occupation and sex. The eCH patients in bout had 1.6 higher odds of rating their health as good or very good compared to cCH patients, but the difference was not significant (OR 1.57, 95%CI 0.96–2.57, *p* = 0.074).

### Impact on social and family life

Overall, being restricted in everyday life was reported by 234 out of 253 eCH patients (92%) during bout and 143 out of 146 (98%) cCH patients. In remission, 37 out of 253 eCH patients (15%) were restricted due to CH.

The majority (71%) of all the participants were married or living with a partner and marital status was evenly distributed among eCH patients, cCH patients, and controls (*p* = 0.104). Although there was an equal distribution of marital status, 18 eCH patients (7%) and 17 cCH patients (12%) reported being single as result of CH. In the eCH population, 54 (21%) described themselves as being dependent on family and friends due to CH compared to 70 (48%) cCH patients.

The decision of becoming a parent was impacted by CH in 16 eCH patients. Of these, 7 patients (3%) completely rejected having children and 9 patients (4%) chose not to have additional children. In the chronic group 11 patients (8%) discarded having children altogether and an additional 21 (14%) limited the number of children due to CH.

Within the last 10 years, 35 eCH patients (14%) and 56 cCH patients (38%) reported reduced ability to live a normal family-life. During a CH attack, the ability to perform normal household activities was reduced by 87% and participation in family social activities was on average reduced by 85%. The effects during the attack was similar among eCH and cCH patients.

### Impact on professional life

In total, 158 CH patients had suffered from CH for less than 10 years and hereof 52 patients (42%) reported that the CH debut had a negative impact on their work-life (Table 2). Among all patients, 157 patients (39%) reported that CH impacted their work life and within the last 10 years 94 patients (24%) lost a job exclusively or partly due to CH. In comparison only 3 (2%) in the control group had lost a job during the last decade. Onset of CH before the age of 20 years did not affect educational level compared to patients with an onset in the second or third decade (Table 3).

**Table 2:** Cluster headache impact on professional life first ten years after debut

	**Episodic cluster headache (*n* = 89)**	**Chronic cluster headache (*N* = 68)**	**All (*N* = 158)**
Changed workplace due to cluster headache	17 (19%)	35 (53%)	52 (33%)
Deselected curtain jobs or assignments due to cluster headache	31 (35%)	34 (50%	65 (41%)
Lost a job due to cluster headache	11 (12%)	22 (32%)	33 (21%)
Disability pension due to cluster headache	3 (3%)	5 (7%)	8 (5%)
Overall, cluster headache impacted the work life	21 (24%)	45 (66%)	52 (42%)

Data presented for the 158 patients who had their first attack within the last 10 years. Data are expressed as number (percentage)

**Table 3:** Education level by age of onset

Cluster headache onset	> 40 years *N* = 122	20–40 years *N* = 190	< 20 years *N* = 88
Age at interview, years	55.8 (6.2)	43.3 (9.86)	39.6 (12.4)
Episodic cluster headache	67 (55%)	129 (68%)	57 (65%)
Cluster headache duration, years	7.7 (5.9)	14.8 (9.5)	25.0 (13.1)
Education level			
Student	2 (2%)	6 (3%)	10 (11%)
Primary/secondary	15 (12%)	21 (11%)	7 (8%)
Technical or associate degree	49 (40%)	85 (45%)	32 (36%)
Bachelor or master degree or higher	50 (41%)	72 (38%)	30 (34%)
Other degree	6 (5%)	5 (3%)	9 (10%)

Data presented for 400 patients as means (SD) for numerical variables and as number (percentages) for categorical variables. The table implies that educational attainment was slightly higher in age of onset above 20 years old. However, the mean age was lower in the group with age of onset before the age of 20 and more people were students. The group only completing primary or secondary level is smallest in the group with onset before the age of 20

In total, 52 patients (13%) were on full time disability pension and hereof 34 (64%) reported that CH was the deciding factor in the application and approval of the disability pension. Altogether, fulltime disability pension was significantly more frequent in cCH patients compared to eCH patients (28 cCH patients (19%) vs 24 eCH patients (9%), *p* = 0.007). Disability pension due to CH, was 5 times more likely for cCH patients compared to eCH patients (*N* = 400, OR 5.0, 95%CI 2.3–10.9, *p* < 0.001) in a logistics regression analysis when correcting for age, gender, highest achieved education and comorbidities (psychiatric and somatic). A bachelor or master’s degree was obtained by 10 patients (29%) out of 34 patients who received disability pension due to CH compared to only 3 patients (16%) out of 19 of patients who received disability due to other disease.

Absence due to sick days within the last year due to CH were recorded by 120 patients (57%) out of the 210 employed patients (full/part-time or self-employed). Among currently employed participants, cCH patients had 31.9 ± 68.5 annual sick days (*N* = 48), eCH patients had 13.6 ± 23.8 (*N* = 165), and controls had 4.1 ± 9.3 (*N* = 200), *p* < 0.001. If excluding CH related absenteeism, cCH patients had 6.9 additional sick days compared to the control group whereas the eCH patients had 1.5 more sick days than controls (*p* = 0.011). Only, 19 patients (9%) had more than 30 annual sick days. Regarding presenteeism, CH patients rated the ability to work as 7.2 ± 2.9 (*N* = 213) on a 11-point rating scale during periods with attacks whereas controls generally rated their productivity at work as 0.7 ± 1.8. Thus, the overall reduction in productivity amounts to 65% in comparison to the control group.

### Direct and indirect cost

The direct and indirect costs are summarized for both eCH and cCH patients in Table 4. In the annual direct cost calculation, cCH patients were on average 6,395 € more costly compared the eCH patients (*p* < 0.001). Acute treatment with triptans and oxygen accounted annually for 3,619 ± 9,100 € per CH patient. Sumatriptan injection was the most expensive acute treatment, and in the group using this treatment, within the last year (*n* = 136) the average expense was 8,106 ± 13,946 € per patient. Whereas oxygen was used by 270 patients within the last year and the average cost here was 803 ± 411 € per patient receiving the treatment.

Another direct cost was the out-patient treatment. Within the last year, 166 patients (42%) had 2.3 ± 2.1 visits in a hospital-based out-patient clinic and 110 patients (28%) reported visits at a practicing neurologist and these patients reported a mean of 2.7 ± 2.6 visits. Additionally, 185 patients (46%) visited their general practitioner regarding CH treatment. Outside office hours, 59 (15%) patients reported visits to the emergency room or an on-call general practitioner for acute consults and herein 10 (17%) patients visited the emergency room more than two times. Within the last year 275 patients received out-patient treatment and the mean out-patient healthcare services amounted to 873 ± 1, 096 €. The mean annual cost of in-patient treatment 6,213 ± 7,227 € but only 55 patients reported hospitalization within the last year Table 4.

**Table 4:** Direct and indirect 1-year cost in € per patient with cluster headache

	**Episodic cluster headache (*n* = 253)**	**Chronic cluster headache (*n* = 146)**	**All (*n* = 400)**
**Direct cost**			
Oxygen, €/year, mean (SD) *Recipients*	297 (317) *N* = *152*	961 (495) *N* = *117*	542 (506) *N* = 270
Triptans, €/year, mean (SD) *Recipients*	1,709 (4,349) *N* = *128*	5,470 (13,467) *N* = 84	3,077 (9,008) *N* = *234*
General practitioners, €/year, mean (SD) *Recipients*	35 (76) *N* = *113*	72 (162) *N* = *72*	49 (116) *N* = *185*
Acute medicine, €/year, mean (SD) *Recipients*	28 (100) *N* = *32*	190 (1229) *N* = *27*	87 (749) *N* = *59*
Neurologist, €/year, mean (SD) *Recipients*	38 (94) *N* = *60*	68 (158) *N* = *50*	49 (122) *N* = *110*
Hospital outpatient clinic, €/year, mean (SD) *Recipients*	352 (738) *N* = *88*	767 (1,186) *N* = *78*	503 (947) *N* = *166*
Hospital admittance, €/year, mean (SD) *Recipients*	303 (12,01) *N* = *24*	1,630 (4,601) *N* = *30*	871 (3,427) *N* = *55*
**Total annual direct cost per patient in €, mean (SD)**	**2,763 (4,741)**	**9,158 (14,237)**	**5,178 (9,980)**
	**Difference: 6,395, *p* < 0.001**		
**Indirect cost**			
Sick days, €/year, mean (SD) *Recipients*	1,337 (3,924) *N* = *90*	5,345 (15,248) *N* = *30*	2,797 (9,898) *N* = *120*
Disability pension, €/year, mean (SD) *Recipients*	2,221 (7,229) *N* = *23*	6,464 (11,384) *N* = *40*	3,764 (9,182) *N* = *63*
**Total annual indirect cost per patient in €, mean (SD)**	**3,558 (7,855) €/year**	**11,809 (17,104)**	**6,561 (1,2696)**
	**Difference: 8,240, *p* < 0.001**		
**Total annual cost per patient in €, mean (SD)**	**6,321 (9,928)**	**20,967 (23,051)**	**11,739 (17,507)**

In the indirect cost calculation, cCH patients were on averages 8,240 € more expensive annually compared to eCH patients (*p* < 0.001).

## Discussion

**Fig. 3:**
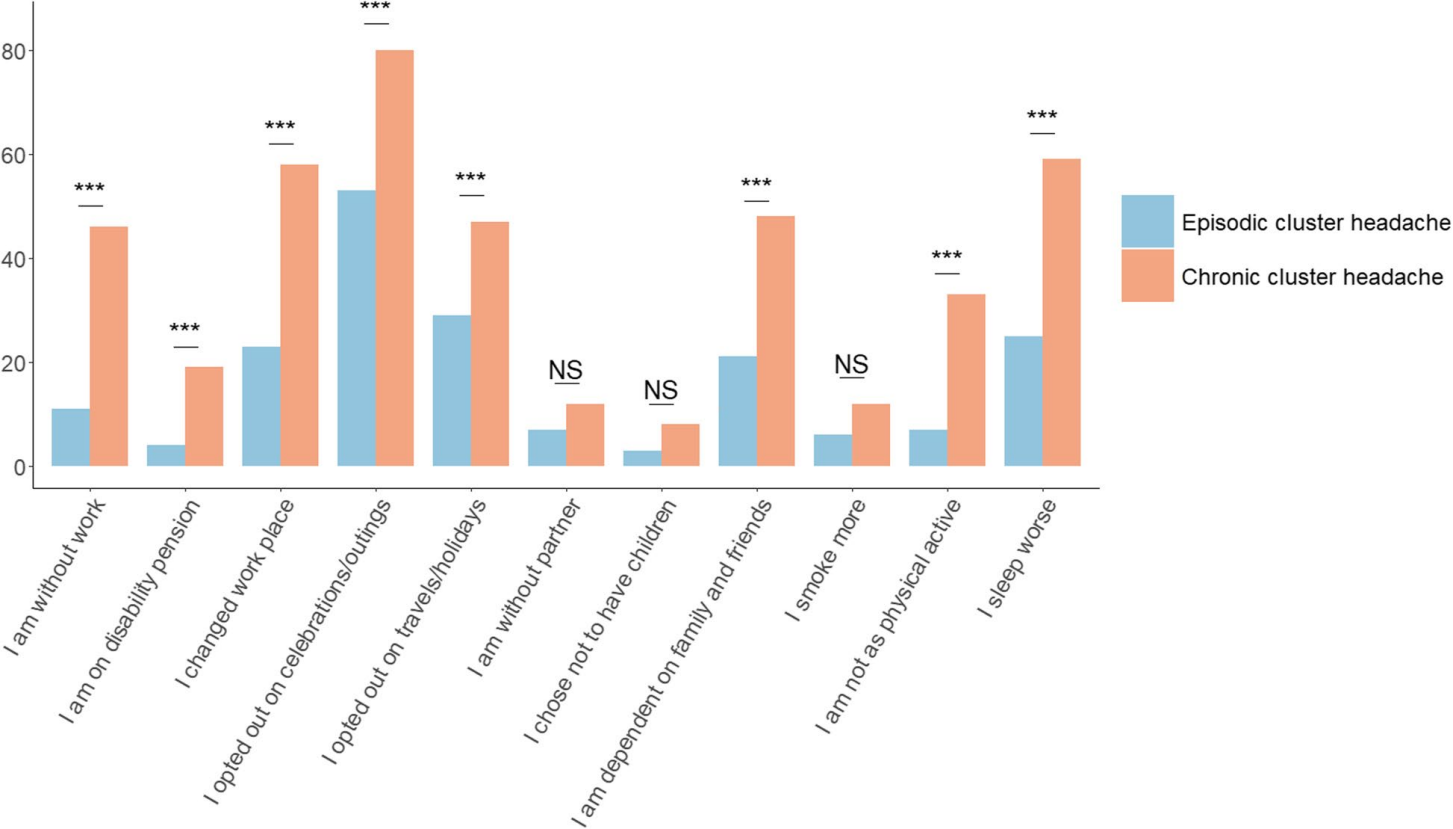
Negative impact of cluster headache on professional, social and family life. Legend: Self-reported impact of cluster headache on different aspects of everyday life. Data presented for 399 chronic and episodic cluster headache patients. Bonferroni correction was applied. *** *p* ≤ 0.001, NS Not significant

CH impacts all investigated aspects of everyday life, especially in the chronic subgroup (Fig. 3). Not unexpectedly, health was rated significantly worse in periods with attacks compared to periods without attacks and to controls, but we could also demonstrate a significant burden for the patients in remission compared to controls. Finally, amongst the 400 CH patients we also identified a mean annual direct cost of 5178 €/patient and substantial loss of productivity resulting in a higher mean annual indirect cost of 6561 €/patient.

Self-rated health was significantly reduced in periods with attacks and cCH patients had ten-fold lower odds of rating their health as good or very good compared to controls. Even eCH patients who had been out of bout for more than a year did not rate their health as good as the controls. Self-rated health is an important predictor and strongly associated with increased mortality [21]. CH have been associated with an increased suicidality [22] and an unhealthy lifestyle increasing the risk of cardiovascular diseases [14]. The combined increased mortality risk should be taken serious by physicians treating CH patients. We unfortunately did not investigate the suicidality in the present study. Self-rated health was in part restored in remission periods but still not equal to the control group. The attack burden, but also comorbidities such as depression, anxiety and suicidality, are eased but still reported during remission [23, 24]. In addition, we found that CH influences many eCH patients when taking important life decisions, such as having (more) children, and their professional life was impacted by job loss and passing up projects at work. The pronounced effect on personal and professional life is in line with previous publications [4, 18, 25, 26]. Therefore, we put forward the argument that eCH, also, is a chronically disabling illness despite the cyclic nature of the bouts, at least for this patient population recruited from a hospital-based headache center. Current preventative is limited by targeting the bout once it occurs whereas the ideal preventative would prevent the occurrence of the bouts. Currently, physicians might reduce the anxiety by making the treatment plan during the remission.

In our cohort, 19% received full/part-time disability pension and this level was expected based on the general distribution for chronic pain patients in Denmark [27]. Full-time disability pension was received by 13% of the CH population and although this is high it is in line with previous findings ranging from 8–15% [18, 28–30]. Of note, the CH population is better educated compared to the general population receiving disability pension, in our cohort 29% had a bachelor or master degree and in the general disability pension population this only accounted for 13% [31]. This was also confirmed by another Scandinavian study, in which the CH diagnosis reduced the effects of education on odds of receiving disability pension [30]. This points to CH causing social drift, rather than social causation. The subgroup with early onset had a stronger affiliation to the job market compared to a later onset. This could indicate that disease severity worsens with later onset; however, this is heavily biased by the Danish social welfare system where it takes time to go through the bureaucratic motions of testing ability to work and hearings. Further, after a reform in 2013 the general rule is that permanent support, such as disability pension, cannot be granted before the age of 40 years. Education level seems relatively unaffected by CH [4] and we demonstrated that this was also true for the patients who debuted young. In patients who debuted in the last 10 years, 21% had experienced a job loss due to CH. In previous studies from Denmark, Italy and the USA, 10–17% of the patients had reported loss of a job due to CH [18, 29, 32] whereas this only occurred in 3% in a recent Korean study [25]. It is worrying that the number of patients losing their job was unchanged from previous publications, however, in our cohort we may have more cCH patients compared to the previous cohorts [29]. New treatment options are highly needed to change the trend of job loss due to CH.

As hypothesized, the described burden is much more pronounced in cCH compared eCH. For example, 11% of the eCH patients indicate that they are without job compared to almost half the cCH patients. Previously, it was found that CH patients had higher employment rates compared to their unaffected family relatives, but this study only included eCH patients[33]. Sick days for employed patients and healthy controls were similar to previous findings in a registry study [34]. Notably, the eCH patients have almost identical number of sick days as controls when excluding CH related sick days but the chronic patients who are employed have twice the amount of sick days. This could be due to higher degree of comorbidities even in the 33% of the chronic population that are still employed.

The direct and indirect cost of CH were high especially in cCH patients where the total cost was three times that of the eCH patients. The direct costs of eCH patients were slightly higher in a German study (1,819 €/half year vs 2,763 €/year) from 2011 [19]. The Italian study from 2020 found a mean cost of 1755 € per bout but since the cost were calculated per bout the findings are not directly comparable to our study. The direct cost of the cCH patients (approximately 9000 €) was similar to the findings by the Italian group [32]. However, the German study found a direct cost of 9073 € within 6 months, but they included high cost procedures as implantation with occipital nerve stimulation and alternative treatments such as massage which were not included in our cost-calculation. The bulk of the cost was attributed to triptans and this is a common denominator in the three studies [19, 32]. Considering the high cost associated with triptans it is important to consider that this cost might be out-weighed by the resultant increased work productivity consequently lowering the indirect cost of CH. Our dataset unfortunately does not allow us to explore this as we were unable to quantify the presenteeism.

The direct cost was in this study based on patient reported outcome, another strategy to investigate this could be via the pharmacy databases in Denmark. Applying different methods of estimating the costs are a strength as they will reduce the common method bias. The indirect cost can be difficult to compare between the studies as there are both methodological and structural differences in social welfare systems. In our analysis we included part- and fulltime disability pensions as an indirect cost as we have no better way to estimate the productivity loss. The imprecisions in the evaluation of the indirect costs are stated as assumptions in the method section. The total indirect cost also includes presenteeism, rehabilitation, the work absence for the spouse and other costs [35]. This is of course a limitation to the final output as generally the indirect cost is hard to quantify. However, similar rates of part-time disability pension was found in another registry study [30]. The Danish welfare system is characterized by high redistribution of income and therefore we believe the human capital approach is the best fit even if it tends to overestimate the cost [36].

Our data is limited due to its cross-sectional nature, self-reports and recruitment from a tertiary headache center. The high rate of CCH patients reflects inclusion primarily form a specialized clinic and the results might not be representative for the entire CH population in Denmark. Another limitation is that we used validated health surveys, and did not investigate the emotional burden, the reason for this was the questionnaire already being quite comprehensive. Also, our control group was more educated than the patient groups and this might bias the health ratings [37]. The strengths being the largest population of validated CH patients diagnosed according to the ICHD-criteria, unbiased to burden alone due to a very broad questionnaire and where both personal and societal burden are explored. It is important to notice that our results have a high variance indicating for some, even a short cluster period may have devastating effects on career and/or family life whereas others are relatively unaffected in their everyday life by cCH. This may depend on attack frequency, treatment response, social network, and education, and still other factors. The treatment response for this population has been published separately [13].

In conclusion, CH affects the patients significantly on both an economic and personal level. The chronic patients are much more affected in everyday life and costly than in the episodic patients. As expected eCH patients are less affected in remission than in bout, however, eCH patients in remission are still limited in important life and career choices. Therefore, we argue that eCH, also, is a chronically disabling illness despite the cyclic nature of the bouts. Hopefully, new emerging treatments will ease this for patients and society.

## Data Availability

The Danish Cluster Headache Survey contains sensitive information and can consequently not be shared in full form according to Danish data protection law. Deidentified data that underlie the results of this article can be shared on request.

## Abbreviations

CH: Cluster Headache
eCH: Episodic cluster Headache
cCH: Chronic Cluster Headache;
OR: Odds ratio
CI: Confidence interval (95%)
